# Isolation and Role of *PmRGL2* in GA-mediated Floral Bud Dormancy Release in Japanese Apricot (*Prunus mume* Siebold et Zucc.)

**DOI:** 10.3389/fpls.2018.00027

**Published:** 2018-01-26

**Authors:** Lin Lv, Ximei Huo, Luhua Wen, Zhihong Gao, Muhammad Khalil-ur-Rehman

**Affiliations:** Laboratory of Fruit Tree Biotechnology, College of Horticulture, Nanjing Agricultural University, Nanjing, China

**Keywords:** DELLA, *RGL2*, gibberellins, dormancy, GA_4_, *SLY1*, Japanese apricot

## Abstract

Bud dormancy release is regulated by gibberellins (GAs). DELLA proteins are highly conserved and act as negative regulators in GA signaling pathway. The present study established a relationship between *PmRGL2* in Japanese apricot and GA_4_ levels during dormancy release of floral buds. Overexpression of *PmRGL2* in poplar delayed the onset of bud dormancy and resulted in dwarf plants, relative to wild-type trees. *PmRGL2* exhibited higher expression during ecodormancy and relatively lower expression during endodormancy. The relative level of GA_4_ exhibited an increasing trend at the transition from endodormancy to ecodormancy and displayed a similar expression pattern of genes related to GA metabolism, *PmGA20ox2*, *PmGA3ox1, PmGID1b*, in both Japanese apricot and transgenic poplar. These results suggests that *PmRGL2* acts as an integrator and negative regulator of dormancy via a GA-signaling pathway. Moreover, an interaction between *RGL2* and SLY1 in a yeast two hybrid (Y2H) system further suggests that SCF E3 ubiquitin ligases, such as *SLY1*, may be a critical factor in the regulation of *RGL2* through an SCF^*SLY1*^-proteasome pathway. Our study demonstrated that *PmRGL2* plays a negative role in bud dormancy release by regulating the GA biosynthetic enzymes, *GA20ox* and *GA3ox1* and the GA receptor, *GID1b*.

## Introduction

Japanese Apricot (*Prunus mume* Siebold et Zucc.), a member of the Rosaceae family, is culturally important deciduous fruit tree (C_3_ plant) in East Asia, including Japan, the Korean peninsula, and southeast China. The fruit is mainly used for making liqueurs, pickles, and sauces (Chuda et al., 1999; Mitani et al., 2013). Bud dormancy is a complex process in perennial plants and allows them to survive seasonal adverse environmental conditions (Baskin and Baskin, 1998) by preventing budbreak and subsequent growth or flowering even during short periods of favorable ambient temperatures (Olsen, 2010). Three types of dormancy have been categorized, paradormancy, endodormancy, and eco-dormancy (Lang et al., 1987), while a fourth stage, dormancy release, has also been proposed (Horvath et al., 2003). (i) Paradormancy is the suppression of lateral budbreak and is regulated by hormonal signals from the terminal shoot meristem (apical dominance); (ii) endodormancy, represents suppression of budbreak by internal bud signals, even under favorable conditions; (iii) ecodormancy, represents inhibition of growth by unfavorable environmental conditions and is negated by warm temperatures. Previous studies have demonstrated that the induction and release of bud dormancy are regulated by hormones (Wareing et al., 1971) and processes related to chilling accumulation (Rinne et al., 2011; Zhuang et al., 2013).

Studies have shown that budbreak in woody angiosperms, as well as other growth-related processes, are dependent on sufficient gibberellin (GA) levels (Saure, 1985; Looney, 1997). Isaia and Bulard (1978) reported that there are significant quantities of free GA_9_, and higher levels of bound GA_4_ in dormant embryos. GA_s_ are also implicated in regulating the timing of the onset of bud dormancy, and the chilling-induced release of bud dormancy. This suggests that while chilling induces GA accumulation in dormant buds, different responses are elicited by GA_3_ and GA_4_ during dormancy release and that bud burst only occurs when sufficient levels of GA_4_ are present (Hazebroek et al., 1993; Schrader et al., 2004; Rinne et al., 2011). *PcGA2ox* deactivates both bioactive GA_1/4_ and their immediate precursors (GA_20/9_) and has a strong dwarfing effect in poplar (Busov et al., 2003) but *PcGA2ox* cannot metabolize GA_3_ (Busov et al., 2006).

Gibberellin levels also have a major impact on GA-metabolism, biosynthesis, and signaling pathways (Sun and Gubler, 2004; Schwechheimer, 2008). GA_4_ (the main bioactive form of GAs) binds to one of its receptors *GIBBERELLIN INSENSITIVE DWARF (GID*) 1A-c, the GA_4_–*GID1* complex interacts with DELLA proteins (Middleton et al., 2012), that are subsequently tagged by the F-box protein, SLEEPY1 (SLY1)-mediated ubiquitin-26S-proteasome, for destruction (Murase et al., 2008). DELLAs act as a negative regulator in GA-dependent growth processes (Nimisha et al., 2013). Arabidopsis (*Arabidopsis thaliana*) has five DELLA proteins: GAI (GAIN SENSITIVE), RGA (REPRESSOR-OF-GA), RGL1 (RGA-LIKE1), RGL2, and RGL3. All of these proteins possess an N-terminal DELLA domain containing the conserved amino acid sequence, Asp-Glu-Leu-Leu-Ala (DELLA), VHYNP motifs, a poly (S/T) region, and a C-terminal GRAS functional domain (Silverstone et al., 1998). Inconsistent GA signaling repression has been reported by high levels of VvDELLA proteins in grape, and grapes exhibit a higher growth response to GA application (Acheampong et al., 2017). Previous studies demonstrated that GAI and RGA play a role – GA-regulation of hypocotyl growth and stem elongation in Arabidopsis (Peng et al., 1997; Dill et al., 2004). *RGL2* plays an essential role in regulating seed germination (Lee et al., 2002). The GAI/RGA-like gene, *RGL2*, is a negative regulator of GA responses that regulate seed germination rather than stem elongation. *RGL2* is an inducible regulatory factor of GA synthesis. The *RGL* mutant, *rgl2*, represses seed germination (Tyler et al., 2004). DELLA proteins act as negative regulators of plant growth. These proteins) can directly regulate the expression of GA biosynthetic enzymes (*GA20ox2* and *GA3ox1*) or GA receptors (*GID1a* and *GID1b*) in GA responsive pathways (Zentella et al., 2007).

The transcriptional repressors of DELLA in Arabidopsis are targets of the SCF complex of F-box proteins SLEEPY (SLY)-1/SNEEZY (SNE)-1. *SLY1* (composed of three main domains: F-box, GGF, and LSL) encodes the F-box subunit of SCF E3 ubiquitin ligase. Studies have demonstrated that SCF^*SLY1*^ positively regulates plant growth by GA signaling. For example, GA-insensitive mutations in *Sleepy1* (*SLY1*) increased seed dormancy and inhibited seed germination in a GA biosynthetic mutant. The *sly1* mutant exhibit a GA-insensitive dwarf phenotype that suppresses the activity of DELLA protein (Steber et al., 1998; Dill et al., 2004; Strader et al., 2004). Previous reports indicate that *SLY1* genes are closely associated with GA signal transduction via targeted DELLA protein RGA (Peng et al., 1997; Dill et al., 2001; Fu et al., 2004; Achard et al., 2009). *GID2* is a positive regulator of a GA-signaling pathway. *GID1*/GA/DELLA complex targets DELLA proteins for degradation via the SCF^*GID2/SLY1*^ proteasome pathway. SLY1, GID2 of SCF E3 ligase complexes target RGA and SLR1 via 26S proteasome (Sasaki et al., 2003; Dill et al., 2004).

The mechanism of dormancy release in Japanese apricot is still poorly understood. Therefore, it is necessary to explore how *RGL2* gene expression affects the regulation of GA metabolic pathways. The purpose of the present study was to investigate identify the *RGL2* gene in Japanese apricot and characterize its expression during different stages of dormancy as a basis for further study. In addition, information on the interaction of PmSLY1 interaction with PmRGL2 was examined by yeast two-hybrid (Y2H) analysis. The overall objective was to determine the relationship between *PmRGL2* and GA_4_ levels during dormancy release.

## Materials and Methods

### Plant Materials and Dormancy Treatments

Floral bud samples were collected from mature trees of Japanese apricot cv. “Taoxingmei” (a low chilling requirement cultivar) located at the National Field Gene Bank for Japanese apricot in Nanjing, Jiangsu Province, China (Gao et al., 2012). Floral buds were collected during four phases of dormancy: paradormancy, prior to leaf fall; endodormancy; ecodormancy; and during the dormancy release (budbreak) period. Samples were collected on September 28, 2015 (paradormancy) (29°/21°C, day/night temperatures), November 2, 2015 (endodormancy) (19°/8°C, day/night temperatures), November 30, 2015 (ecodormancy) (13°C/5°C, day/night temperatures), and January 12, 2016 (dormancy release) (8°C/-1°C, day/night). The sampling was carried out as described by Wen et al. (2016). All tissues were immediately frozen in liquid nitrogen and stored at -70°C until further use. Three independent lines of transgenic poplar and wild-type trees (*Populus tremula* × *Populus alba*) were placed in an environmental chamber set at low temperature conditions for approximately 3 months to observe their growth (8.5°C/4°C, 16 h light/8 h dark).

### RNA Extraction and Reverse Transcription – Quantitative PCR (RT-qPCR)

Total RNA was isolated from flower buds of Japanese apricot and frozen leaves of transgenic wild-type poplar, using PrimeScript^TM^ RT reagent Kit with gDNA Eraser (TaKaRa, China) reagent according to the manufacturer’s protocol. First strand synthesis of cDNA was carried out using a PrimeScript^TM^ II Ist Strand cDNA Synthesis Kit (TaKaRa, China) following the manufacturer’s protocol. RT-qPCR was carried out using SYBR Premix Ex Taq^TM^ (TakaRa, China). Expression of *PmRGL2* (KJ667048), *Pm20ox2* (XM_008234605.2), *Pm3ox1* (XM_008244481.2), *PmGID1b* (XM_008236735.1) and RNA polymerase II (*RP II*) (XM_008238347.2) were examined in apricot bud samples at different stages of dormancy (**Supplementary Table S1**).

Transgenic poplar was also subjected to RT-qPCR. RNA samples were isolated from tissue culture plants. Expression levels of *PmRGL2* (KJ667048), *PpGA20ox2* (XM_011043353.1), *PpGA3ox1* (XM_011041681.1), *PpGID1b* (XM_011011170.1), and *EFIα* (GQ253565.1) were examined by RT-qPCR. All RT-qPCR analyses were repeated three times. *RPII* (XM_008238347.2) and *EFIα* (GQ253565.1) were used as internal controls for Japanese apricot and poplar samples, respectively (Tong et al., 2009; Su et al., 2013). PCR amplification reactions were performed using Power SYBR Green PCR Master Mix (TaKaRa, Japan) in a Step One^TM^ Real-Time PCR System. The reaction mixture (20 μl) included 1 μl of diluted cDNA (equivalent to 100 pg of total RNA), 4 pmol of each primer, and 10 μl of Power 2×SYBR Green PCR master mix (SYBR Green RT-qPCR Master Mix; TaKaRa, Japan). The PCR protocol was as follows: 95°C for 3 min; 40 cycles at 95°C for 20 s, 60°C for 20 s, and 72°C for 40 s. The 2^-ΔΔC_T_^ method was used to estimate relative expression level (Livak and Schmittgen, 2001).

### Gene Isolation and Bioinformatic Analysis

Total RNA extraction from mixed floral and vegetative buds and subsequent cDNA synthesis were carried out from samples collected at the four different dormancy stages previously described. RT-PCR was performed with primers designed using Primer 5 software, based on the sequence of *Pm014329* (XM_008230973) (**Supplementary Table S1**). A total volume of 25 μl, containing 100 ng of cDNA, 5 mM dNTP mixture, 100 μ mM of each primer, 0.625 U of PrimeSTAR GXL DNA polymerase (TaKaRa Biotechnology, Dalian, China), and 5 μl 5X PrimeSTAR GXL buffer (Mg^2+^ plus) was used in PCR amplification. The PCR reaction consisted of 35 cycles (30 s at 94°C, 40 s at 59.6°C, 2 min at 72°C). A 1,794-bp product was purified and cloned into pEASY–Blunt Cloning vector using a pEASY–Blunt Cloning Kit (TransGen Biotech, Beijing, China). The obtained sequence was blasted and its homology with *RGL2* sequences in other plant species was confirmed. This Japanese apricot sequence was designated as *PmRGL2* (KJ667048) (**Supplementary Table S1**). A multiple alignment of the deduced amino acid sequence was performed with sequences from different species using ClustalW and GeneDoc software, and a phylogenetic tree was constructed using the neighbor-joining method in MEGA 5.0. The statistical reliability of the phylogenetic tree was determined by bootstrap analysis with 1,000 replicates. The basic physical and chemical properties of the proteins were predicted by Expert Protein Analysis System^1^. NCBI^2^ was used to detect conserved domains using default parameters.

### Liquid Chromatography–Tandem Mass Spectrometry (LC–MS/MS)

Frozen tissues (flower buds 100 mg, transgenic leaves 1 g) were extracted in dark conditions at 4°C, filtered, and mixed with 5 ml 80% methanol and containing an internal standard (10 μg) and sonicated for 10 min (Fernández et al., 2003; Durgbanshi et al., 2005). The extract was purified using a C18 Sep-pack column (6 ml/500 mg, United States) to remove the pigments and eluted with 5 ml 60% methanol and then freeze-dried. The dried samples were then dissolved in 1 ml methanol and stored at -20°C until further use. The samples were injected into a chromatogram column (4.6 mm × 100 mm, 18 μm) at 40°C at a flow rate of 0.4 ml/min. Mobile phase A, consisting of 0.1% methanoic acid, and mobile phase B, consisting of 100% acetonitrile, were used for chromatographic separation. Initial conditions were 60% A and 40% B which was maintained for 4 min, changing linearly to 5% A, 95% B over 16 min, 0% A, 100% B for 15 min, and finally maintained at 60% A, 40% B for 18 min. The conditions of mass spectrometry were as follows: ESI spray voltage, 4 kV; sheath gas flow-rate, 70 arb; auxiliary gas flow-rate, 20 arb; capillary temperature, 350°C and tube lens, 95 V. GA_4_ was monitored at m/z transitions of 331→213. The optimized collision energy for GA_4_ was 20 eV.

The LC–MS/MS conditions used for the Japanese apricot floral bud samples were as follows: ESI spray voltage, 4 kV; sheath gas flow-rate, 70 arb; auxiliary gas flow-rate, 20 arb; capillary temperature, 350°C and tube lens, 95 V. GA_4_ were monitored at m/z transitions of 331→213. The optimized collision energy for GA_4_ was 20 eV. The LC–MS/MS conditions for the transgenic poplar chromatography and mass spectrometry procedures were as described above with a slight modification. The transitions monitored were *m*/*z* 331.5 for GA_4_ and *m*/*z* 121.02 for benzoic acid (internal standard). A minor peak occurred at 8.889 min and 4.782 min for the GA_4_ and benzoic acid standard, respectively.

### Construction of *PmRGL2* Overexpression Vector and Plant Transformation

The full-length coding sequence of *RGL2* was amplified by PCR. The *PmRGL2* CD_S_ fragment was fused to a β-glucuronidase (GUS) gene, replacing the 35S CaMV (Cauliflower mosaic virus) promoter in the pYH4215 vector. Overexpression vectors were introduced into poplar using a leaf disk transformation method (Horsch et al., 1985). *In vitro* transgenic and non-transgenic plantlets were transferred to pots filled containing potting soil with nutrients and moved to an environmental chamber (24°C, 16 h light/8 h dark) to adapt external environmental. After 2 weeks, transgenic and non-transgenic plants were put the chamber room for dormancy. *PmRGL2* positive transgenic plants were determined by RT-PCR. Three independent transgenic poplar lines (T_1_, T_2_, T_3_) were used in subsequent RT-qPCR experiments. The three *PmRGL2*-overexpression transgenic poplar lines were also used for phenotypic analysis during different stages of dormancy. Transgenic trees were compared to wild-type trees.

### Yeast Two-Hybrid Assay

A yeast two-hybrid assay was used to determine if PmRGL2 could interact with PmSLY1 (Genbank accession no. XM_008237725, gene data not shown). The full-length CDs of *PmRGL2* and *PmSLY1* were amplified by PCR with gene-specific primers and a bait vector (pGBKT7-PmRGL2) and prey vector (pGADT7-PmSLY1) were constructed (**Supplementary Table S1**). These constructs were transformed into Y2H Gold cells following the manufacturer’s protocol (Clontech). Self-activation and toxicity detection of the recombinant plasmid and control vectors (pGADT7-T, pGBKT7-53, and pGBKT7-Lam) were carried out as described in the Matchmaker^TM^ Gold Y2H manual. Cultured yeast cells re-suspended in YPDA were plated on selective DDO media and incubated at 30°C for 4 days. Yeast cultures containing either the interactor vector (pGADT7-PmSLY1+pGBKT7-PmRGL2), positive vector (pGADT7-T+pGBKT7-53), or negative vector (pGADT7-T+pGBKT7-Lam) were all placed on DDO, DDO/A, QDO, QDO/A/X. In order to check for any false or positive interactions, yeast cells containing an empty “prey” vector and an empty “bait” vector were co-transformed with interactor clones and plated as above. Single colonies were selected and patched on DDO, DDO/A, QDO, and QDO/X/A media while interactions were selected at 30°C for 3 days. Positive colonies were confirmed by PCR.

### Statistical Analysis

Analysis of variance (ANOVA) was used to compare statistical differences in dormancy treatments and levels of gene expression at different dormancy stage between Japanese apricot and transgenic plants. Differences between test samples were determined using a Duncan’s multiple range test at a significance level of *P* ≤ 0.05. Three technical replicates were used for each biological replicate, and the data shown represent the mean ± standard errors (SE; *n* = 3). Three biological replicates were used for each of the genotypes, the wild type, Y2H.

## Results

### Isolation and Characterization of *PmRGL2*

**FIGURE 1:**
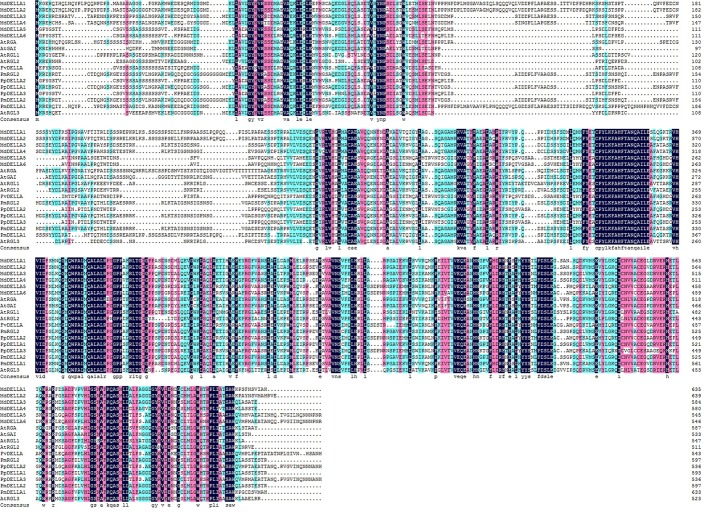
Amino acid sequence alignment of *PmRGL2* and DELLA proteins from other plant species, including: *Malus domestica*, ACL68360.1; *Malus hupehensis*, ABS50250.1; *Vitis vinifera*, AAM19210.1; *Rosa lucieae*, AFC88482.1; *Pyrus × bretschneideri*, AFJ23220.1; *Arabidopsis thaliana*, NP_186995.1; *Prunus mume*, XP_008229195.1; and *Prunus persica*, XP_007214956.1; *PmRGL2*, KJ667048.

**FIGURE 2:**
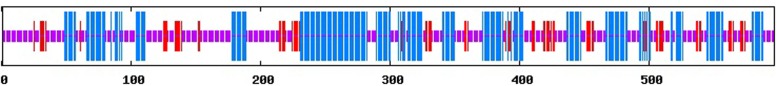
Prediction secondary structure of PmRGL2. Blue: random coil, purple: α-helix, red: extended strand.

**FIGURE 3:**
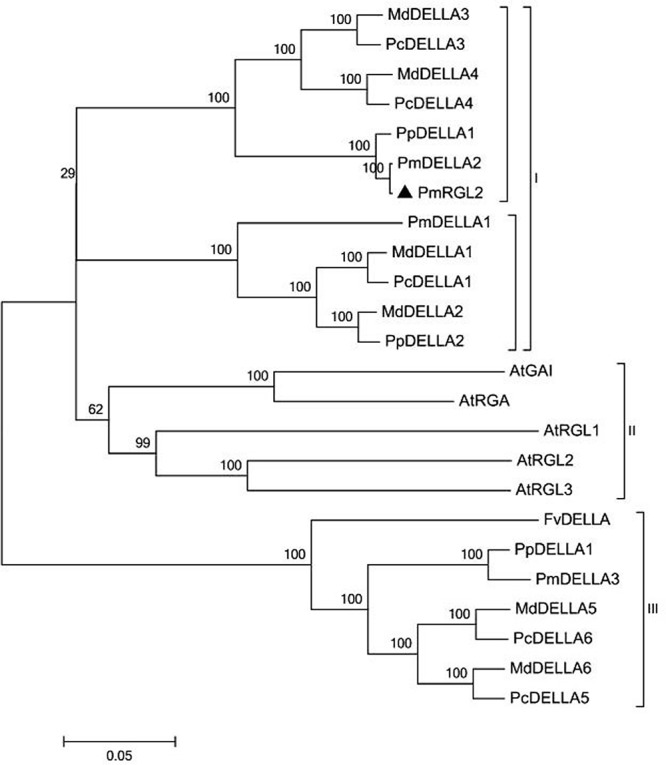
A phylogenetic tree of PmRGL2 from Japanese apricot and DELLA proteins from other plant species, including: *Malus domestica*, ACL68360.1; *Malus hupehensis*, ABS50250.1; *Vitis vinifera*, AAM19210.1; *Rosa lucieae*, AFC88482.1; *Pyrus × bretschneideri*, AFJ23220.1; *Arabidopsis thaliana*, NP_186995.1; *Prunus mume*, XP_008229195.1; and *Prunus persica*, XP_007214956.1; *PmRGL2*, KJ667048.

Sequence analysis demonstrated that *PmRGL2* clone obtained was a full-length sequence with a complete open reading frame (ORF). *PmRGL2* encodes an *RGL2* protein of 1,794 bp amino acid residues with a putative molecular mass of 64 kDa, and an isoelectric point (IP) of 5.05. Further analysis indicated that *PmRGL2* displays its highest identity with a DELLA protein in *Prunus persica* (XM_007214894) by BLAST (**Figure 1**). Therefore, the obtained clone was designated *PmRGL2* (*Prunus mume RGL2*). The secondary structure of *PmRGL2* was predicted using Prabi^3^ (**Figure 2**). The analysis predicted that 39.2% of the amino acids were in α-helix, 47.4% in random coil, and 13.4% in an extended strand. Analysis of conserved motifs revealed that PmRGL2 protein possesses two signature DELLA and TVHYNP motifs, that define the DELLA protein subfamily. The relationship between *PmRGL2* and other DELLA proteins was assessed by constructing a phylogenetic tree (**Figure 3**) using the complete amino acid sequences of PmRGL2 and DELLA proteins sequence from other species. The DELLA proteins clustered into three groups, designated I, II, and III. Only the DELLA protein from *Fragaria vesca* fell into group III. The DELLA protein from *Arabidopsis thaliana* clustered into group I. PmRGL2 and PmDELLA2 clustered in the group I. In addition, the same species in DELLA proteins consistent of the same group-II, such as AtGAI and AtRGA.

### GA_4_ Levels and *PmRGL2* Expression in Floral Buds

**FIGURE 4:**
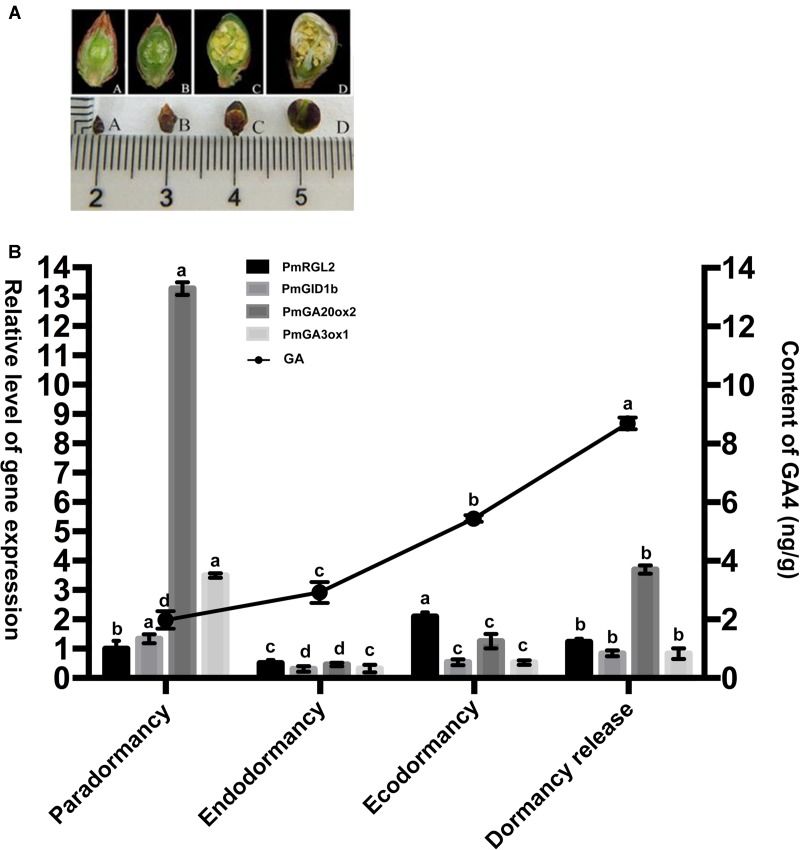
Expression of *GA20ox2*, *GA3ox1*, and *GID1b* in Japanese apricot is influenced by the expression of *PmRGL2* during the different stages of dormancy. **(A)** Series of lateral flower buds sampled during the different stages of dormancy. (A) Paradormancy before leaf fall, (B) endodormancy, (C) ecodormancy, (D) dormancy release. **(B)** GA_4_ levels in Japanese apricot determined by LC-MS/MS and the expression level of *PmRGL2, GA20ox2*, *GA3ox1*, and *GID1b* in floral buds of Japanese apricot during of the four stages of dormancy as determined by RT-qPCR. Small letters over a column indicate significant differences at *P* ≤ 0.05 (Duncan’s multiple range test). Data represent the mean of three biological replicates where each biological replicate consisted of three technical replicates.

The relationship between the pattern of expression of *PmRGL2* (as determined by RT-qPCR) and GA_4_ levels (as determined by LC–MS/MS) were analyzed to better understand the role of *PmRGL2* in dormancy release (**Figure 4** and **Supplementary Figure S1**, respectively). During the sample collection, morphological changes in flower buds during different dormancy stages were evaluated by observing dissected bud under a stereomicroscope.

Flower buds were relative thin during paradormancy and stamens were produced during this time period. The lowest GA_4_ levels were also recorded during paradormancy. Maximum GA_4_ levels and low levels of *PmRGL2* expression were observed when flower buds began to swell (**Figures 4A,B**). Expression of *PmRGL2* was highest during ecodormancy. The concentration of GA_4_ in flower buds increased slightly during endodormancy, relative to the level observed during paradormancy, while a trend of elevated GA_4_ levels was observed during ecodormancy and dormancy release. The expression of *PmGA20ox2*, *PmGA3ox1*, *PmGID1b* were up regulated during ecodormancy, relative to para- and endodormancy. The expression of *PmRGL2* decreased during dormancy release (**Figure 4B**). Therefore, it was concluded that the expression of *PmRGL2* is negatively correlated with changes in GA_4_ levels, suggesting that *PmRGL2* may have an inhibiting effect on dormancy release.

### Over Expression of *PmRGL2* in Poplar Inhibits Plant Growth

**FIGURE 5:**
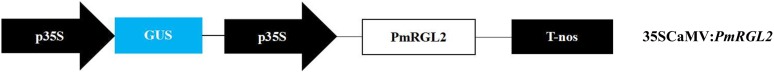
The *PmRGL2* overexpression construct. The *PmRGL2* gene was cloned and inserted in the pYH4215 vector. *PmRGL2* expression was driven by the 35S CaMV promoter.

**FIGURE 6:**
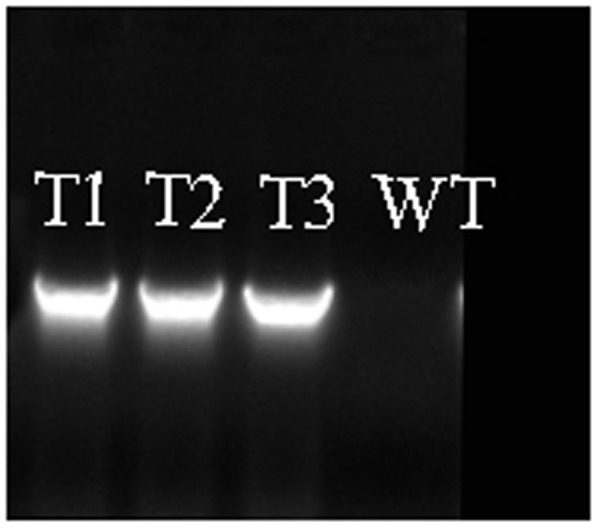
Confirmation of *PmRGL2* in three, independent lines of transgenic poplar (T_1_, T_2_, T_3_), and its absence in wild-type poplar lines. WT, wild-type.

**FIGURE 7:**
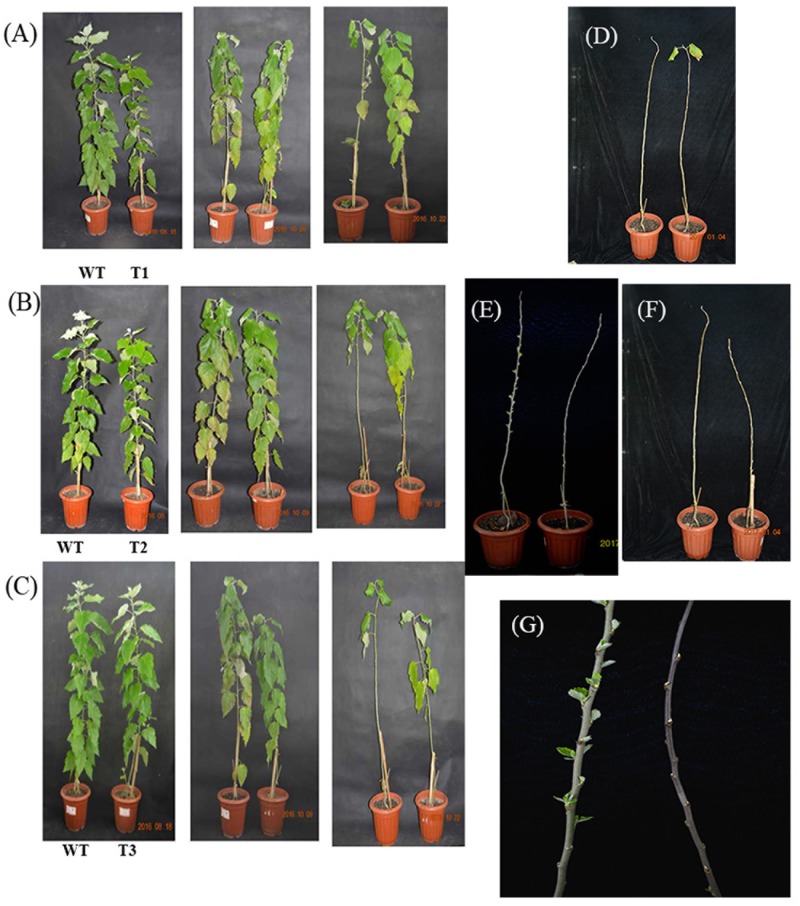
Phenotypic characterization of transgenic poplar plants constitutively expressing *PmRGL2* in comparison with non-transgenic, wild-type, poplar plants. A, B, C are different transgenic lines. WT, wild-type. Plant height (measured from soil line to tip of flag leaf): WT: 90 cm, T_1_: 88 cm, T_2_: 81 cm, T_3_: 78 cm. Wild-type on the left and transgenic lines on the right in each figure. In the four pictures of each group, photos were taken after 0, 52, 70, and 83 days after entering into dormancy. The WT is on the left in each picture **(A–F)**, and the transgenic tree is on the right. T_1_ **(A,D)**, T_2_ **(B,E)**, T_3_ **(C,G)**. **(G)**, close-up of buds in WT (left) and transgenic poplar (right) during dormancy release.

**FIGURE 8:**
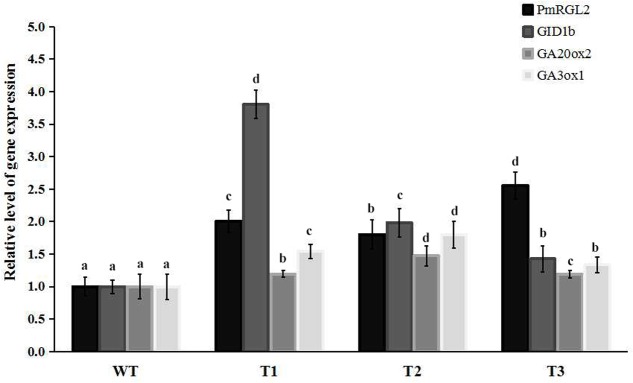
Relative expression levels of *PmRGL2*, *GID1b, GA20ox2*, and *GA3ox1* in poplars and as measured by RT-qPCR. Different letters over the columns indicate a significant difference at *P* ≤ 0.05 (Duncan’s multiple range test). Data are the mean ± SD (*n* = 3).

**FIGURE 9:**
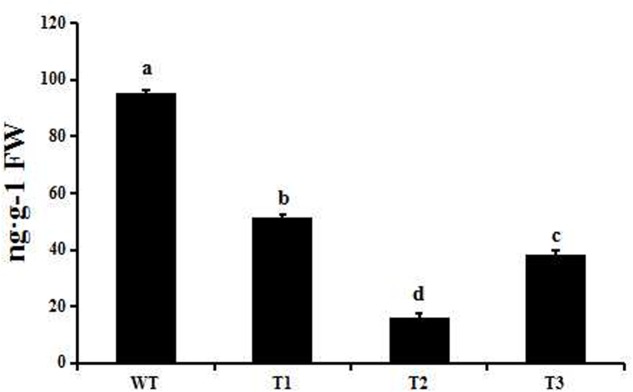
GA_4_ levels in transgenic and wild-type poplar as measured by LC-MS/MS. Small letters over a column indicate significant differences between the WT and transgenic lines at *P* ≤ 0.05 (Duncan’s multiple range test). Data are the mean ± SD (*n* = 3).

Transgenic poplar plants constitutively overexpressing *PmRGL2* were to evaluate the role of *PmRGL2* gene in GA responsiveness, A 35S CaMV:*PmRGL2* construct (**Figure 5**), was used to transform in poplar leaves. Three independent transgenic lines (T_1_, T_2_, T_3_) were obtained and *PmRGL2* expression levels were examined in each of the transgenic poplar lines by RT-qPCR using *PmRGL2* gene-specific primers. All three transgenic lines exhibited high levels of *PmRGL2* expression and were used in subsequent analyses. *PmRGL2* transcripts were not detected in the non-transformed wild type poplar trees (**Figure 6**). While the transgenic and non-transgenic trees were in the environmental chamber it was observed that all of the lines of the transgenic poplar plants grew slowly (**Figures 7A–C**), relative to wild-type trees. When plants entered into dormancy, the expression level of *PmRGL2* decreased in the buds of the transgenic plants. During the dormancy release stage, budbreak in the wild-type trees occurred earlier than in the transgenic plants (**Figures 7D–G**). These data and the observed phenotypes suggest that *PmRGL2* may function as an integrator in the regulation of GA biosynthesis and metabolism. The expression levels of GA-related genes and *PmRGL2* were analyzed in transgenic and non-transgenic plants to confirm if GA receptor expression is associated with *PmRGL2* regulation of GA biosynthesis. Total RNA was extracted from *PmRGL2* transgenic and wild-type plantlets grown on a tissue culture medium at 24°C (16 h light/8 h dark) and analyzed by RT-qPCR. Results indicated that expression of *PpGID1b, PpGA20ox2*, and *PpGA3ox* were more highly transgenic plants, relative to expression levels in non-transgenic plants (**Figure 8**). GA_4_ levels in leaves of *PmRGL2* transgenic and wild-type plantlets were also assessed. LC-MS/MS analysis indicated that the concentration of GA_4_ was slightly lower in plantlets overexpressing *PmRGL2* relative to the GA_4_ level in wild-type plantlets (**Figure 9**). These data indicate that overexpression of *PmRGL2* results in a lower level of GA in transgenic poplar plants.

### Yeast Two-Hybrid Analysis of the Interaction between PmRGL2 and PmSLY1

**FIGURE 10:**
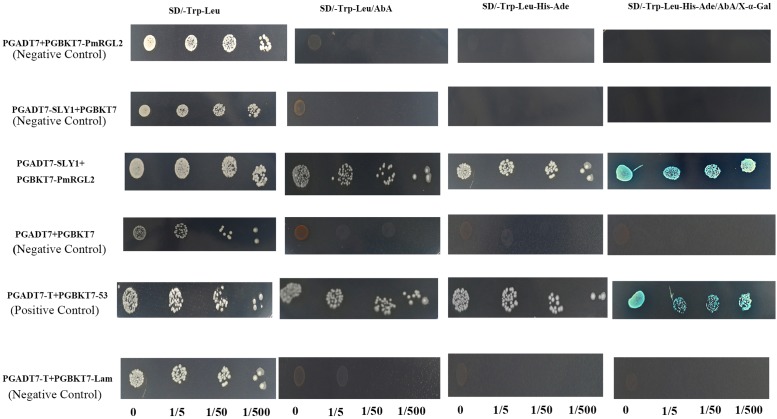
Interaction of *RGL2* with SLY1 in a yeast two-hybrid assay. Three dilutions (0, 1/5, 1/50, and 1/500) of yeast cultures adjusted to an OD_*600*_ of 0.4 were spotted on synthetic medium to maintain proper growth. Photos were taken after 4 days of culture. Experiments were performed three times, and each experiment contained at least three replicates.

The interaction between *RGL2* and SLY1 was assessed using a yeast two-hybrid assay, where SLY1 was fused to a binding domain (DB), and *RGL2* was fused to an activation domain (AD). Results demonstrated that *RGL2* has a protein-protein interaction with SLY1 (**Figure 10**). Thus, SCF E3 ubiquitin ligase may regulate the *RGL2* DELLA protein in Japanese apricot where∖SLY1 regulates GA signaling during dormancy stages.

## Discussion

Silverstone et al. (1998) reported five DELLA genes (*RGA*, *GAI*, *RGL1*, *RGL2*, and *RGL*) in *Arabidopsis thaliana*. These genes have also been reported in other plant species, and designated as *d8* in maize (*Zea mays*), *SLN1* in barley (*Hordeum vulgare*), *VvGAI* in grape (*Vitis vinifera*), *SLR1* in rice (*Oryza sativa*), and *AhDELLA* in peanut (*Arachis hypogaea*) (Boss and Thomas, 2002; Muangprom et al., 2005; Foster et al., 2007; Lawit et al., 2010; An et al., 2015). Previous reports have indicated that DELLA proteins play an important role in dormancy release and other processes, such as stem elongation, plant height, dormancy, seed germination, and floral and root development (Koornneef et al., 1984; Peng and Harberd, 1993; Silverstone et al., 1998; Peng et al., 1999; Dill and Sun, 2001; Dill et al., 2001; Nakajima et al., 2001; Lee et al., 2002). Although *RGL2* studies have been conducted, their regulatory role in GA signaling is poorly understood, especially in non-model organisms. Therefore, the function of the *RGL2* DELLA protein was investigated in Japanese apricot in regard to its role in dormancy.

Multiple sequences alignment of DELLA proteins from several species, including PmRGL2 from Japanese apricot, indicated that they are highly conserved. Proteins within the DELLA subgroup sharing these conserved motifs are likely to have similar functions in the GA-signaling pathway (Hussain and Peng, 2003; Sato et al., 2014). DELLA subfamily proteins interact with the GA-binding receptor, *GID1*, thus playing a critical role in perceiving GA signals to regulate plant development (Murase et al., 2008). Deletion of 108 residues in the N-domain, encompassing the conserved DELLA and TVHYNP motifs, inhibits RGA degradation (Fuentes et al., 2012). Sequence differences between *PmRGL2* and other DELLA proteins may reflect functional adaption or functional differentiation that occurred over the course of species evolution. GA binding to *GID1* results in the formation of a GA-GID1-DELLA complex that weakens the inhibitory effect of DELLA proteins on plant growth. And DELLA proteins also form a DELLA ubiquitin E3 ligase complex (SCF^*SLY1/GID2*^) which targets DELLA protein for degradation by the 26S proteasome (Ariizumi et al., 2011; Hauvermale et al., 2014). DELLA proteins, such as RGL2, negatively regulate GA response (Ikeda et al., 2001; Hussain and Peng, 2003). Recently, Middleton et al. (2012) also used molecular modeling to study feedback loops in GA signaling. In the present study, an *RGL2* gene was isolated from Japanese aprico*t. PmRGL2* possesses both DELLA and GRAS domains with a comparison of PmRGL2 with other DELLA proteins (**Figures 2**, **3**) revealed that the DELLA protein domains in *PmRGL2* are similar to the DELLA domains in other species, possessing DELLA, TVHYNP, VHIID, RKVATYFAEALARR, RVER, and SAW domains. The presence of these structural domains in PmRGL2 protein confirms that it is a member of the DELLA family of proteins. Some of the amino acid sequences in PmRGL2 were different than other DELLA proteins (NP_178266 and NP_172945) suggesting that functional differences may be present.

Transcript levels of the GA biosynthetic genes *GA20ox* and *GA3ox* are upregulated when the GA catabolic gene, *GA2ox*, is down regulated (Sun and Gubler, 2004). In our study, GA_4_ content increased in Japanese apricot buds over the course of the dormant period and reached a peak during dormancy release. Flower buds were observed to be thin and stamens appeared during paradormancy, while the level of GA_4_ during the same time period was low and expression of *PmRGL2*, *PmGA20ox2*, *PmGA3ox1*, and *PmGID1b* expression was high. Swelling of the floral buds and GA_4_ content gradually increased during endodormancy, while expression of *PmRGL2* decreased of the downregulation of *PmRGL2* was co-incident with the downregulation of *GA20ox2*, *GA3ox1*, and *GID1b* (**Figure 4B**). These findings are consistent with a previous study conducted by Middleton et al. (2012). During ecodormancy, GA_4_ binds to the GID1 receptor, and this complex binds to DELLA proteins, and there is an accumulation of the GA_4_–GID1-DELLA (RGL2) complex which induces the biosynthesis of GA_4_. GA_4_ subsequently mediates the expression of *GA20ox2*, *GA3ox1*, and *GID1b*. During dormancy release GA_4_ levels are high and *RGL2* is downregulated (Middleton et al., 2012). DELLA proteins mediate the transcription of *GA20ox2*, *GA3ox1*, and *GID1b* and at the same time repress the transcription of DELLA genes. Dormancy-induced growth suppression decreases with the decrease in the expression of *RGL2* and *GA20ox2*, *GA3ox1*, and *GID1b* expression increases during dormancy release. Transgenic poplar plants constitutively expressing *PmRGL2* exhibited lower levels of native, poplar *GID1b*, *GA20ox2* and *GA3ox1*, relative to non-transgenic wild-type poplar plants (**Figure 8**). Previous studies have reported that GA catabolism by *GA2ox2* decreases over the course of the dormant period while GA synthesis, such as *GA3ox1*, increase (Footitt et al., 2011). Xiao et al. (2010) reported that overexpression of *GhGA20ox1* in transgenic plants enhanced GA production and promoted elongation of fiber cells. The current study provides evidence that *PmRGL2* regulates the expression of *GID1b*, *GA20ox2*, and *GA3ox1*, as well as GA_4_ levels. The *GID1b*, *GA20ox2* and *GA3ox1* were all upregulated in transgenic poplar plants expressing *PmRGL2*, relative to wild-type plants, and GA_4_ levels were lower. Middleton et al. (2012) reported that *GA20ox2*, *GA3ox1*, and *GID1a* expression is downregulated by a GA_4_ treatment. DELLAs are involved in maintaining GA homeostasis through feedback that upregulates the expression of GA biosynthesis and receptor genes (Zhang et al., 2010). All DELLA-regulated genes, including GA biosynthetic enzyme genes and GA receptor genes, are repressed by GA and activated by DELLAs (Zentella et al., 2007).

The expression pattern of *PmSLY1* was similar to *PmRGL2* during the different stages of dormancy (**Supplementary Figure S2**). Acheampong et al. (2017) reported that decreased expression of *VvSLY1b* may be responsible for inducing a massive accumulation of VvDELLA proteins, which then led to elevated *VvGID1* levels. The SLY1 homolog, SLY2 (SNE), directly binds to RGA proteins, suggesting that it negatively regulates a subset of DELLA proteins regulated by SLY1. Overexpression of *SLY2* can rescue dwarfism and infertility in *sly1–10* mutants by reducing the accumulation of DELLA proteins, RGA, and GA (Fu et al., 2004; Strader et al., 2004; Ariizumi et al., 2011). Previous studies demonstrated that RGL1 and RGL3 exhibit a weak interaction with SLY1 and that in contrast sly1-d interacts more strongly with RGL1, RGL2, and RGL3 than does SLY1. RGA (DELLA protein) and GAI directly interact with SLY1 in Y2H assays and *sly1-d* has an influence on the C-terminal region of SLY1 that enhances the interaction between RGA and GAI (Dill et al., 2004; Tyler et al., 2004). The loss-of-function *sly1* mutant and *GID2* mutant are GA-insensitive dwarfs (Sasaki et al., 2003). High levels of *RGL2* expression are observed in *sly1* mutants after GA treatment and its repression of seed germination is inactivated after-ripening in *sly1* mutant seeds (Ariizumi and Steber, 2007). In the present study, lower levels of *PmRGL2* expression were reflected by lower *SLY1* levels, and constitutive expression of *PmRGL2* in transgenic poplar plants resulted in a dwarf phenotype over (**Figure 7**). These results further indicate that *RGL2* acts as a regulator of GA homeostasis and transcript levels of GA biosynthetic genes.

**FIGURE 11:**
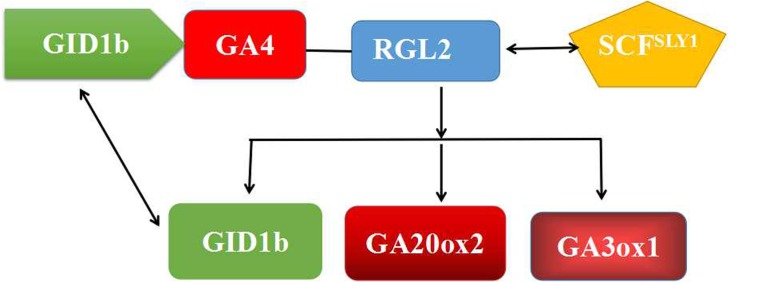
A simple model representing the role of *RGL2* and GA_4_ in the regulation of bud dormancy by regulating the expression of GA-related genes. Under dormant conditions, RGL2–SCF^*SLY1*^ complex regulate its expression and thereby inhibits dormancy in buds. Degradation of *RGL2* and expressionist repression of GA related-genes results in dormancy release.

The *SLY1* gene is closely associated with GA signal transduction by targeted DELLA protein RGA (Peng et al., 1997; Dill et al., 2001; Fu et al., 2004; Achard et al., 2009). PmRGL2 interacted with PmSLY1 in the Y2H assay (**Figure 10**), which supports the premise that SLY1 targets *RGL2* for degradation in response to GA (Dill et al., 2004). Our collective data demonstrate that *PmRGL2* is a DELLA protein gene that is upregulated during endodormancy in Japanese apricot. *RGL2* isolated from Japanese apricot acted as a negative regulator of the GA signaling pathway. Constitutive expression of *RGL2* in transgenic poplar plants delayed budbreak but did not slow down the rate of leaf senescence in the fall, and resulted in a dwarf, GA-deficient phenotype. The Y2H assay provided evidence supporting a specific interaction between PmRGL2 and PmSLY1, thus indicating that SLY1 targets the DELLA protein, RGL2, for degradation. Our results indicate that *RGL2* acts as a repressor of GA responses and also acts as a negative regulator of GA-promoted budbreak (**Figure 11**). *RGL2* plays a pivotal role in the regulation of GA responses. The GA/DELLA interaction is critical to the regulatory network controlling plant growth. Additional studies will be required to elucidate the function of other PmDELLA proteins in dormancy and other growth processes regulated by GA.

## Conclusion

Our study provided insight into the role of *PmRGL2*, *GID1b*, *GA20ox2*, and *GA3ox1* in GA signal transduction during dormancy release of floral buds in Japanese apricot. Constitutive expression of *PmRGL2* in transgenic poplar plants exhibited delayed budbreak. GA levels increased when the GA biosynthetic genes, *GA20ox2* and *GA3ox1*, were upregulated and the expression of the gibberellin receptor gene, *GID1b*, was downregulated. Further studies will help to determine all of the mechanisms underlying GA-mediated bud dormancy and allow for the identification of additional dormancy proteins that are targeted by SLY1.

## Author Contributions

Conceived and designed the study: ZG, LL, and XH. Performed the experiments: LL, XH, and LW. Wrote the manuscript: LL. MK-u-R helped in revising and editing the manuscript. Data analysis: LL, XH, and LW. All authors read and approved the final version of the manuscript.

## Conflict of Interest Statement

The authors declare that the research was conducted in the absence of any commercial or financial relationships that could be construed as a potential conflict of interest.

## Footnotes

1 http://expasy.org/proteomics

2 http://blast.ncbi.nlm.nih.gov/Blast.cgi

3 https://npsa-prabi.ibcp.fr/cgi-bin/npsa_automat.pl?page=/NPSA/npsa_hnn.html

## Supplementary Material

FIGURE S1 A typical LC-MS/MS chromatogram of a standard solution containing 5 μg mL^-1^ GA_4_ **(A,B)**. Values are the mean ± SD (*n* = 3).

FIGURE S2 Relative expression of *PmSLY1* at different stages of dormancy. Different letters indicate significant differences (*P* ≤ 0.05) between the different stages of dormancy as determined by a Duncan’s multiple range test. Data represent the mean ± SD (*n* = 3).

TABLE S1 Primer sequences used for cloning, expression analysis, vector construction, and confirmation of transformation.
