# Supplementary figures and images for: Isolation and Role of PmRGL2 in GA-mediated Floral Bud Dormancy Release in Japanese Apricot (Prunus mume Siebold et Zucc.)
Source: Front Plant Sci. 2018 Jan 26;9:27. doi: 10.3389/fpls.2018.00027 (PMC5790987; doi:10.3389/fpls.2018.00027)

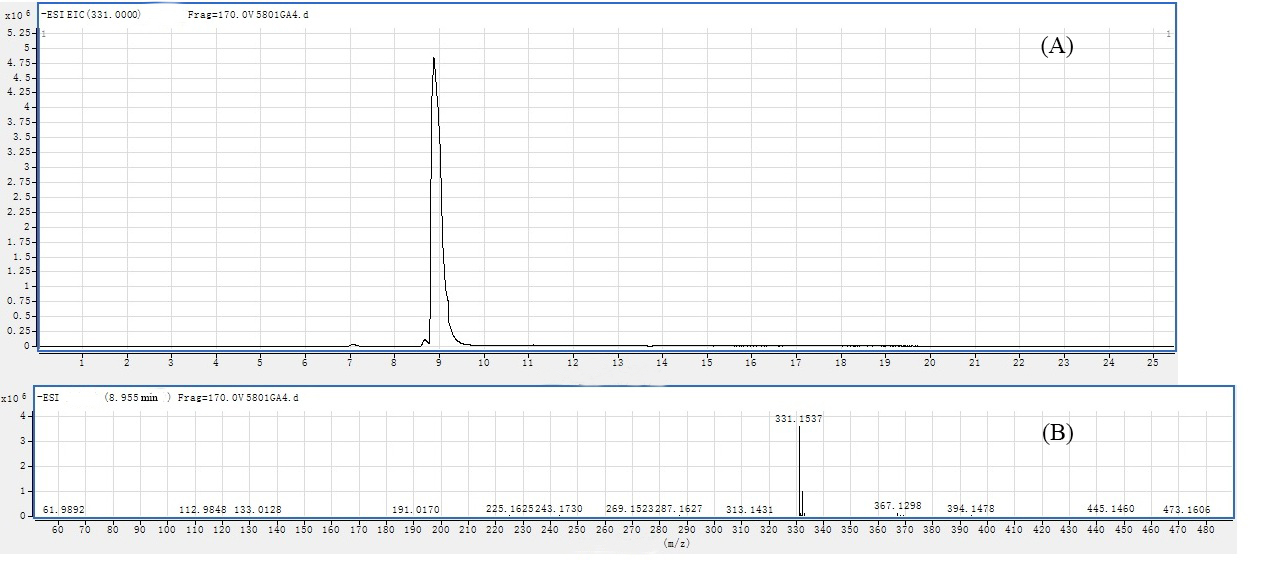

Supplement: FIGURE S1 — A typical LC-MS/MS chromatogram of a standard solution containing 5 μg mL-1 GA4 (A,B). Values are the mean ± SD (n = 3). [file Image_1.JPEG]

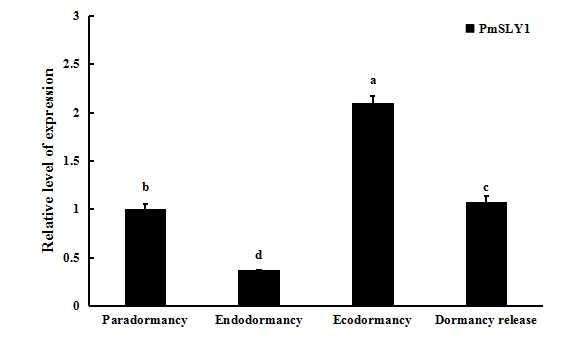

Supplement: FIGURE S2 — Relative expression of PmSLY1 at different stages of dormancy. Different letters indicate significant differences (P ≤ 0.05) between the different stages of dormancy as determined by a Duncan’s multiple range test. Data represent the mean ± SD (n = 3). [file Image_2.JPEG]
