# Supplementary material for: Isolation and Role of PmRGL2 in GA-mediated Floral Bud Dormancy Release in Japanese Apricot (Prunus mume Siebold et Zucc.)
Source: Front Plant Sci. 2018 Jan 26;9:27. doi: 10.3389/fpls.2018.00027 (PMC5790987; doi:10.3389/fpls.2018.00027)
Supplement: TABLE S1 — Primer sequences used for cloning, expression analysis, vector construction, and confirmation of transformation. [file Table_1.DOCX]

**Table S1 Primer sequences used for cloning, expression analysis, vector construction, and transgenic confirmation .**

| **Genes** | **Accession No.** | **Sequences (5ʹ-3ʹ)** | |
| --- | --- | --- | --- |
|  |  | **Forward** | **Reverse** |
| *PmRGL2* | KJ667048 | CTTCCTCAATCACCTCCATA | GTTTTCAATCGCTTTCTCCT |
| *Pm20ox2* | [XM_008234605.2](http://www.ncbi.nlm.nih.gov/entrez/viewer.fcgi?db=nucleotide&id=1027104254) | TGAACTACTATCCCAGATGC | ATCAGGCTTAGGAGTAACAG |
| *Pm3ox1* | [XM_008244481.2](http://www.ncbi.nlm.nih.gov/entrez/viewer.fcgi?db=nucleotide&id=1027113764) | CAGTGAACTCAATCATGCAG | GAACATGGTCGAGTAGTAGTA |
| *PmGID1b* | [XM_008236735.1](https://www.ncbi.nlm.nih.gov/entrez/viewer.fcgi?db=nucleotide&id=645258595) | TATCCTTGTGCGTATGATGA | TTCCCAATACCTCAACTTCT |
| *RPII* | [XM_008238347.2](http://www.ncbi.nlm.nih.gov/entrez/viewer.fcgi?db=nucleotide&id=1027106972) | TGAAGCATACACCTATGATGATGAAG | CTTTGACAGCACCAGTAGATTCC |
| *PpGA20ox2* | [XM_011043353.1](http://www.ncbi.nlm.nih.gov/entrez/viewer.fcgi?db=nucleotide&id=743896768) | CGACACCAGACAAACATAC | ACATGCTTCACCAACAAG |
| *PpGA3ox1* | [XM_011041681.1](http://www.ncbi.nlm.nih.gov/entrez/viewer.fcgi?db=nucleotide&id=743893498) | CCTGCTCCATATCCTATCAA | GCGACAAGGTATTCATTCC |
| *PpGID1b* | [XM_011011170.1](http://www.ncbi.nlm.nih.gov/entrez/viewer.fcgi?db=nucleotide&id=743930447) | ACAGAGAACACAATCAGAGA | GTCCTTCAACATAAGCCAAT |
| *EFIɑ* | GQ253565.1 | ATTGACAGGCGGTCTGGTAAGGAA | AAACGACCAAGTGGAGGATACTCT |
| *Pm014329* | XM_008230973 | ATGAAGAGAGATCACCGGGATACCT | TCACCGGGTTGACTCAGTCGAACTC |
| *RGL2* | KJ667048 | CGGGATCCATGAAGAGAGATCACCG (BamHI site is underlined) | CGAGCTCTCACCGGGTTGACTCAGT (SacI site is underlined) |
| *YPmRGL2* | KJ667048 | GGAATTCCATATGATGAAGAGAGATCACCGGGA | CGGGATCCTCACCGGGTTGACTCAGT |
| *YPmRGL2* | KJ667048 | GGAATTCCATATGATGAAGAGAGATCACCGGGA | CGGGATCCTCACCGGGTTGACTCAGT |
| YPmSLY1 | XM_008237725 | GGAATTCCATATGATGGCTCTCAATCTGCG (NdeI site is underlined) | CGGGATCCTCACTGAGTTGAAGCAGCAGG (BamHI site is underlined) |
| pGADT7 |  | TAATACGACTCACTATAGGG | AGATGGTGCACGATGCACAG |
| pGBKT7 |  | TAATACGACTCACTATAG GG | TAAGAGTCACTTTAAAATTTGTATAC |
